# Budgeting for Environmental Health Services in Healthcare Facilities: A Ten-Step Model for Planning and Costing

**DOI:** 10.3390/ijerph17062075

**Published:** 2020-03-20

**Authors:** Darcy M. Anderson, Ryan Cronk, Lucy Best, Mark Radin, Hayley Schram, J. Wren Tracy, Jamie Bartram

**Affiliations:** 1 The Water Institute, Gillings School of Global Public Health, University of North Carolina at Chapel Hill, Chapel Hill, NC 27599, USA; rcronk@live.unc.edu (R.C.); lucylandis@gmail.com (L.B.); mradin@unc.edu (M.R.); schramh@live.unc.edu (H.S.); jwren@email.unc.edu (J.W.T.); jbartram@email.unc.edu (J.B.); 2 School of Civil Engineering, University of Leeds, Leeds LS2 9JT, UK

**Keywords:** healthcare facilities, environmental health, water, sanitation, and hygiene, WaSH, waste management, cleaning, infection prevention and control, costing, budgeting

## Abstract

Environmental health services (EHS) in healthcare facilities (HCFs) are critical for safe care provision, yet their availability in low- and middle-income countries is low. A poor understanding of costs hinders progress towards adequate provision. Methods are inconsistent and poorly documented in costing literature, suggesting opportunities to improve evidence. The goal of this research was to develop a model to guide budgeting for EHS in HCFs. Based on 47 studies selected through a systematic review, we identified discrete budgeting steps, developed codes to define each step, and ordered steps into a model. We identified good practices based on a review of additional selected guidelines for costing EHS and HCFs. Our model comprises ten steps in three phases: planning, data collection, and synthesis. Costing-stakeholders define the costing purpose, relevant EHS, and cost scope; assess the EHS delivery context; develop a costing plan; and identify data sources (planning). Stakeholders then execute their costing plan and evaluate the data quality (data collection). Finally, stakeholders calculate costs and disseminate findings (synthesis). We present three hypothetical costing examples and discuss good practices, including using costing frameworks, selecting appropriate indicators to measure the quantity and quality of EHS, and iterating planning and data collection to select appropriate costing approaches and identify data gaps.

## 1. Introduction

Maintaining a hygienic healthcare environment is critical for safe care provision in healthcare facilities (HCFs). Inadequate environmental conditions can reduce care seeking [1] and increase the risk of healthcare-acquired infections (HAIs) [2,3]. HAIs are estimated to affect 15% of all hospitalized patients in low- and middle-income countries (LMICs) [4,5] and are the leading cause of death among hospitalized patients [6]. Approximately 60%–80% of HAIs are attributable to unsafe environmental conditions and inadequate healthcare worker hygiene [7].

Environmental health services (EHS) in HCFs are services that prevent contamination of the healthcare environment—and by extension HAIs—and allow for functional care delivery [8]. The specific services considered to be EHS vary across disciplines. Sustainable Development Goal (SDG) 6 sets targets for achieving universal access to water, sanitation, and hygiene (WaSH) by 2030. The Joint Monitoring Program for Water Supply, Sanitation, and Hygiene (JMP) of the World Health Organization (WHO) and UNICEF, which monitors SDG 6 progress, interprets universal access to include HCFs. The JMP monitors water, sanitation, hygiene, medical waste management, and surface cleaning in HCFs [9]. Additional EHS, such as laundry and vector control, are recognized by the WHO, but not included under JMP monitoring [8].

According to the most recent JMP data, 26% of HCFs lack access to basic water, 22% lack basic sanitation, 16% lack handwashing facilities, and 40% do not follow proper waste segregation procedures [10,11]. Data for cleaning and waste management more broadly are planned, but not yet available. Other studies suggest that 73% of HCFs lack sterilization equipment [12].

A poor understanding of the costs of EHS delivery hinders progress toward adequate provision, particularly in LMICs. Only 22% of countries have budgets in place for EHS in HCFs that are consistently funded [13], and understanding the costs of delivery has been identified as an important research need [14]. However, a systematic review rated a majority of existing studies on the costs of EHS in HCFs as poor quality, in part due to the incomplete disaggregation and reporting of environmental expenses, infrequent and inconsistent reporting of unit costs comparable across facilities, and limited contextual data on the quality and quantity of services achieved per money spent [15].

Tools specifically designed to cost EHS in HCFs in LMICs are lacking. Tools exist for costing in non-HCF settings (e.g., community-based [16] or school-based [17] WaSH), but these tools are inadequate for capturing the specific needs of EHS in HCFs. Similarly, healthcare economics methods and tools exist, but are better suited to cost healthcare outputs, such as costs per dialysis session [18,19]. These methods are poorly suited to cost EHS, which are generally delivered at the facility level and are challenging to disaggregate at the patient or procedure level. Furthermore, costing EHS requires knowledge from diverse staff in an HCF who may not typically directly communicate, including accountants, healthcare providers, cleaners, and maintenance workers. Models specifically designed for EHS in HCFs would address challenges of existing tools and provide a common language for the collaboration of a diverse range of costing-stakeholders [15].

Our goal in this study is to develop a process model to guide costing-stakeholders through budgeting for EHS in a range of HCF settings. Our model describes ten steps necessary for the planning, data collection, and synthesis of cost and non-cost contextual information required for budgeting. In this paper, we present our ten-step model; discuss actions for each step; and review existing tools, approaches, and good practices for budgeting. We distinguish between “costing” as a process of collecting and calculating information on the costs of EHS delivery versus “budgeting” as a process of estimating anticipated expenses in a specific context and time period, often for the purpose of appropriately allocating resources. Budgeting uses cost data, but also requires information on the EHS delivery context and goals to inform appropriate planning.

## 2. Materials and Methods

We developed our model based on a review of 47 studies that costed EHS in HCFs. The data source for these studies was a systematic review on the costs of providing EHS in HCFs. The method for the systematic review is described elsewhere [15]. Briefly, the search was designed to identify studies that assessed the costs of establishing, operating, and/or maintaining EHS in HCFs in LMICs and covered five databases (PubMed, EBSCO Global Health and Business Source Premier, Scopus, Web of Science, and ProQuest Theses and Dissertations) from inception through 24 September, 2019. Bibliographic information for each of the 47 studies is provided in Supplement 1.

Two authors (D.M.A. and L.B.) reviewed the sections of each paper that described the costing or budgeting methodology, identified discrete steps in the budgeting process, and developed inductive codes and definitions to categorize and describe each step. Steps were ordered into a preliminary model, which was revised twice to generate the final model presented here. In the first round of revision, the same two authors iteratively re-reviewed and coded all studies using steps defined in the preliminary model. Where the methods described aligned poorly with the preliminary model, we redefined, added, or removed steps as necessary, to improve the model fit. In the second round of revision, all authors reviewed the model to further refine the definitions and order of model steps and to generate a list of actions to be taken for each step. Second-round revisions were informed by approximately 1000 hours of field experience across all authors in 12 countries in the World Bank regions for Latin America and the Caribbean, Middle East and North Africa, and Sub-Saharan Africa. For selected published examples, see [20,21,22,23].

Our discussion on good practices for costing is informed by studies included in model development and the authors’ field experience. We identified good practices of studies that scored highly in risk of bias assessments compared to studies with low scores (see [15] for details). Additionally, we reviewed selected guidelines for EHS delivery in HCFs [8,24,25,26,27], costing studies of EHS from non-HCF contexts [23,28,29], guidelines for costing healthcare and HCFs [18,30], and reporting standards for costing [31,32].

## 3. Results and Discussion

### 3.1. Costing-Stakeholders

Budgeting requires a knowledge of operations and maintenance procedures for delivering EHS, resource inputs for EHS delivery and corresponding outputs, mechanisms for procuring resources, accounting systems and any relevant records keeping practices, and funding streams. A single individual is unlikely to hold all relevant knowledge, and costing-stakeholders should therefore assemble diverse teams. At the facility level, stakeholders for costing include, at a minimum, funders; facility administrators; facility staff responsible for accounting, procurement, and EHS delivery; and facility staff with knowledge of how EHS are provided and used within the facility, such as cleaners, maintenance workers, and healthcare providers. Where facilities have multiple funding mechanisms, stakeholders may include a mixture of private, governmental, and non-governmental organizations. Additional stakeholders may need to be included to secure administrative or ethical permission.

Costing-stakeholders do not need formal education or training to be included. For example, maintenance workers or cleaners with little or no formal education can provide essential information on the maintenance needs of EHS infrastructure. However, certain applications of cost data may require individuals with formal training. For example, costing-stakeholders should include formally trained economists or experienced policy makers if data are to be applied in cost-effectiveness research or setting health systems’ budgets, respectively. Similarly, costing-stakeholders who wish to assess costs of complying with a new national policy on infection control should include an expert who is familiar with the policy.

### 3.2. Model Overview

**Figure 1:**
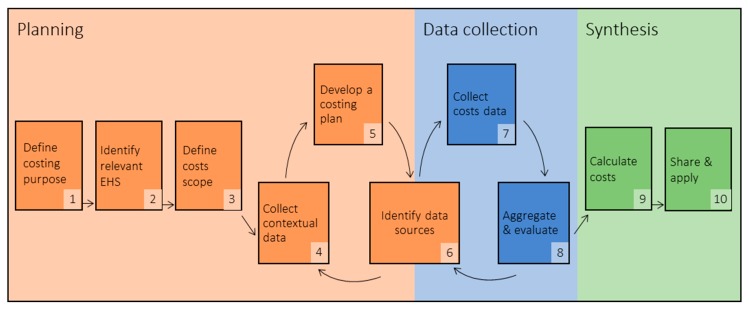
A process model for costing environmental health services (EHS) in healthcare facilities.

Our model describes ten steps organized into three phases: planning, data collection, and synthesis. A process model is depicted in Figure 1. Actions for each step are described in Table 1.

In the planning phase, costing-stakeholders define the purpose of costing, identify which EHS will be costed, and define the scope of costs to be included in cost calculations (steps 1–3). Stakeholders must then understand the context in which EHS will be provided, gathering data on EHS quantity (i.e., demand for or amount of EHS provided), EHS quality (e.g., safety, reliability, and accessibility), and inputs and outputs necessary for EHS delivery (step 4). These definitions and contextual assessment inform the development of a costing plan (step 5), which identifies the approach that will be used for data collection. Stakeholders then identify data sources to gather cost information (step 6). Steps 4–6 of the planning phase are iterative. If facilities selected for data collection do not fit the context of target outcomes, or anticipated data sources are inadequate or unavailable, costing-stakeholders may need to iterate steps 4–6.

In the data collection phase, costing-stakeholders execute their costing plan (step 7), and then aggregate data collected across all sources and evaluate their quality and completeness (step 8). The data collection phase may also require iteration. If aggregation and evaluation indicate information gaps, stakeholders need to collect additional data and possibly identify new data sources. In some instances, for example, if data sources that are suited to the selected costing approach are unavailable, data gaps for critical information may require stakeholders to begin the planning phase again.

Finally, in the synthesis phase, costs are calculated in relevant units (step 9) and information is disseminated to stakeholders and applied for decision making (step 10).

Considerations for each step are described below.

### 3.3. Step 1: Define Costing Purpose

#### 3.3.1. Time and Resources Required for Budgeting

The time and resources available will directly influence the level of detail of information that can be gathered, and costing-stakeholders should consider this when defining a costing purpose. The time and resources needed for data collection will depend on the approach used, level of detail required, timeframe selected, and extent to which desired information is already captured by existing health information management systems. In the studies we reviewed to develop this model, we found data collection periods ranging from several days to over a year.

Budgeting that is informed by retrospective data collection will be less resource-intensive up-front. Where EHS costs are disaggregated from other expenses and already readily available in information management systems, budgeting may conceivably be done in under a month. However, if data require intensive processing to disaggregate EHS costs, budgeting may require several weeks or even months to disaggregate detailed information on line-item expenses. Prospective studies will typically require more time for data collection, but can reduce the resources required for data processing, as costing-stakeholders can collect only relevant information.

Short timeframes of days or weeks for data collection may be appropriate for scenarios where EHS demand is relatively consistent, such as personal protective equipment costs in a clinic with low variability in the day-to-day patient volume. For example, Danchaivijitr et al. [33] measured personal protective equipment use on two days representative of a typical weekday and the holiday patient volume to extrapolate annual costs. However, longer timeframes of months or years will likely be necessary to capture expenses which are infrequent, but may be substantial, such as major repairs to infrastructure. In these cases, prospective data collection may not be feasible, and costing-stakeholders should consider an alternative approach. For example, experienced contractors may be able to give high-level estimates for the installation and annual maintenance expenses of a borehole in key informant interviews over the course of a single day, but these estimates will likely lack detail on specific line-items and may have high uncertainty. Ultimately, costing-stakeholders must weigh the tradeoff between time and resources required for data collection, level of detail, and uncertainty before proceeding with subsequent steps in this model.

#### 3.3.2. Defining Target Outcomes, Target Facilities, and Timeframes

The intended use of cost data will dictate the type of data that must be collected and appropriate methods for collecting them. Costing-stakeholders should agree on the purpose of costing and identify how findings will be applied prior to data collection. Once costing-stakeholders agree on the costing purpose, they must define the health- or EHS-related outcomes targeted by spending (e.g., reduce HAIs or meet JMP indicators for basic sanitation, hereafter referred to as “target outcomes”) and the facilities in which these outcomes are to be achieved (hereafter referred to as “target facilities”). Finally, costing-stakeholders must identify the timeframe over which target outcomes are to be achieved in target facilities. Setting specific targets helps improve healthcare quality management and performance outcomes [34].

Two purposes for costing information are common. First, cost information may inform budgets for planned or existing EHS, where the EHS is typically identified before costing begins. For example, a health clinic may need to the budget for safe water provision. In this instance, target outcomes are related to EHS provision, such as compliance with national water quality standards or ensuring 24-hour access.

We propose three common budgeting needs relevant to EHS in HCFs. Table 2 provides three corresponding hypothetical examples of steps in the planning phase. In Example 1, facilities budget for the operations and maintenance of existing EHS. These facilities already provide adequate EHS, for which the target outcome is to sustain services. Example 2 describes facilities with the target outcome of upgrading or rehabilitating existing services. These may be facilities with inadequate services or facilities with adequate but aging infrastructure planning for future major rehabilitation expenses. In Example 3, facilities budget for the installation of new services where no EHS currently exists.

These examples are not mutually exclusive. A facility can, and often should, budget to operate and maintain existing services while simultaneously budgeting for future major rehabilitations [35]. Similarly, when installing new technology, facilities should budget for future operations and maintenance. Budget shortfalls for repairs are a common cause of service delivery gaps [36]. Different stakeholders may fill different budgeting roles. For example, a non-governmental organization may finance the costs of EHS installation, while the facility is responsible for funding subsequent operations and maintenance.

In particular, when budgeting for new services, costing-stakeholders may need to cost only some components of an EHS. For instance, in our hypothetical Example 3, a non-governmental organization budgets for the installation of a new borehole and solar pump, which connects to existing mechanisms for storage and distribution within the facility that are already covered under existing budgets. However, costing-stakeholders should carefully justify excluding any costs to ensure that data do not underestimate true costs and are fit-for-purpose.

In the second purpose of costing, cost information may also be used as a decision-making tool to prioritize investments. For example, a health system may wish to reduce maternal mortality or HAIs, and cost-effectiveness studies can inform the selection of EHS which are best suited to meet these goals. In these cases, target outcomes are not EHS-specific, but rather focus on health outcomes. Cost data are just one necessary input for cost-effectiveness or cost-benefit studies and must be combined with other data that quantify the effectiveness or benefits. Methods to quantify EHS effectiveness and benefits are beyond the scope of this study, but we direct the reader towards existing examples [37,38,39], and guidelines for data collection [18,40,41] and reporting [31]. In addition, a systematic review and meta-analysis may assist with this by comparing studies of different EHS.

### 3.4. Step 2: Identify Relevant EHS

#### 3.4.1. Defining EHS in HCFs

Consistent, widely accepted definitions for the scope of environmental health in HCFs are lacking. The JMP monitors “WaSH in HCFs” indicators for water, sanitation, and hygiene, but extends beyond the normal scope of “WaSH” to include waste disposal and environmental cleaning [43]. Guidelines from the WHO published in 2008 identify “essential environmental services” for HCFs in LMICs, including JMP WaSH in HCF indicators and environmental services for laundry, food hygiene, wastewater management, and vector control [8]. Within medical disciplines, “standard precautions” and “infection control and prevention” include practices for hand hygiene, protective equipment use, waste handling, and cleaning designed to protect patients and providers [44]. Furthermore, many EHS depend on systems which have no direct environmental outputs, but are essential for routine EHS operation, such as the requirement for power and electricity to operate infrastructure [45].

Despite differences in definitions and categorizations across disciplines, these services share a common theme. At their core, EHS are services that are designed to prevent contamination in the healthcare environment. EHS prevent contamination from person-to-person (e.g., through the use of gloves during care provision) and person-to-environment, and vice versa (e.g., through the use of safe sanitation facilities). EHS protect not only patients and providers, but also non-medical staff (e.g., cleaners and waste handlers), patient caregivers, and communities living in the surrounding environment (e.g., households near waste disposal sites or sewer outlets).

#### 3.4.2. Defining EHS Modalities and Levels

Adequate EHS may be provided through multiple modalities. For example, safe sanitation facilities may include pit latrines where waste is composted in-situ or toilets connected to a municipal piped sewer network, both of which meet WHO guidelines for safely isolating feces from human contact [43]. When budgeting for the installation of new EHS or upgrades, administrators must consider which modalities of EHS provision are fit-for-purpose. EHS modalities that are feasible and appropriate for small, rural clinics providing outpatient services may not be suitable for large, urban in-patient facilities, and vice versa.

When budgeting for EHS in HCFs, administrators must consider the level at which services are to be provided in target facilities. For example, the JMP defines levels of “none,” “limited,” “basic,” and “advanced” services [11]. Higher levels are associated with an improved quality and safety, but also additional costs, such as costs of water quality testing at the advanced service level [10,46]. Upgrading or rehabilitating existing services to higher levels is a relevant concern for facilities where services are present but inadequate and will have different associated costs compared to facilities where no services are available.

### 3.5. Step 3: Define the Scope of Relevant Costs

The scope of costs comprises the type and timeframe of expenses. EHS costs typically comprise both “hardware” (i.e., infrastructure, equipment, and other physical goods) and “software” (training and non-tangible goods, such as procurement, licensing, and insurance) expenses. We propose nine categories of expenses relevant to costing EHS in HCFs (Table 3). Defining and sorting costs into categories helps ensure that all relevant costs are included.

The timeframe may be considered in the context of life-cycle cost analysis (LCA). LCA considers the lifespan of hardware from installation to operations and maintenance to decommissioning and disposal [47]. It accounts for the uneven distribution of costs over time [48]. In relation to the three examples described in Table 2, Example 1 primarily considers the costs of operations and maintenance, Example 2 considers all phases of the lifecycle, and Example 3 primarily considers installation costs. Some EHS may have low installation costs compared to lifetime operations and maintenance costs [35]. While LCA is typically used for hardware costing, the concept is relevant to software costs. For example, software costs required to orient staff to new technologies immediately after installation will differ from costs for routine refresher trainings.

LCA ensures that costing-stakeholders appropriately consider long-term costs and weigh potential trade-offs between upfront installation versus long-term operations and maintenance costs. Similarly, LCA can safeguard against donors or other external funding agencies installing EHS without considering long-term operations and maintenance, and in which the HCFs themselves cannot fund those costs. During budgeting, costing-stakeholders should select and justify cost categories and timeframes that are relevant to their target outcomes. The exclusion of certain expenses may be appropriate in certain circumstances but should be justified. For example, district hospitals may not need to budget for capital hardware if these costs are covered through a separate funding stream at the national level—instead, national budgets should account for these costs. The inappropriate exclusion of cost types or timeframes of a service’s lifecycle will lead to underestimates of budget needs [18].

### 3.6. Step 4: Collect Non-Cost Contextual Data

Before collecting cost data, costing-stakeholders must understand the context in which EHS will be provided. Costing-stakeholders should conduct an assessment in target facilities that considers both general characteristics of the facilities, such as the type of medical services provided, number of patients served, and number of medical professionals on staff, as well as the quantity and quality of EHS. The facility’s location and its geographic proximity to markets are also important cost drivers (e.g., for transportation costs of goods) [18] that should be assessed in this step. These contextual data provide the necessary foundation for all future steps in the budgeting process, as they ensure that facilities meet expected criteria for inclusion, ensure that all requisite cost data are considered, and allow for the adequate disaggregation of cost data.

EHS quantity is the number of EHS needed to meet the patient demand, such as liters of water needed per day for care delivery. EHS quantity is driven in large part by the number of patients and type of services they receive, although evidence suggests that the correlation between the EHS quantity demand and patient volume is not simply linear [39,49,50,51]. EHS quality is the safety and adequacy of an EHS for safe care delivery, such as the microbial and chemical quality of water. EHS quality needs will vary based on the specific procedure and the healthcare services provided at a given facility. For example, water quality needs for surface cleaning in patient waiting areas will differ from those for drinking water.

EHS quality and quantity can be measured directly or assessed through indicators. Direct measures assess EHS inputs or outputs, such as the volume of waste generated or number of sterile gloves used. Indicators assess facility characteristics as proxies for EHS inputs or outputs. For example, floor area is a commonly used indicator for demand for cleaning [52].

Where possible, established indicators and questions should be used, such as those from the USAID Service Provision Assessment [53], the WHO Service Availability and Readiness Assessment [54], and the JMP core questions and indicators for WaSH in HCFs [11]. Using established indicators and questions promotes the comparability of cost data across facilities and therefore facilitates the consolidation of cost data to determine general costs of EHS delivery as opposed to costs for a single facility.

In some cases, additional contextual data may be needed, depending on the costing purpose established in step 1. For example, if compliance with national standards is a key target outcome, indicators and measurement procedures to assess compliance with national standards should be added to this step. If costing-stakeholders require detailed information on specific line-items contributing to overall costs, additional data on the specific inputs and outputs used in EHS delivery are necessary to contextualize these costs. Established indicators provide a solid foundation for collecting quantitative measures of EHS delivery, but do not include the qualitative process knowledge required to understand the way that environmental health services are provided and thereby develop a comprehensive costing plan. Mixed methods data collection can be a useful strategy to collect quantitative measures of established indicators and qualitative measures for context-specific targets.

### 3.7. Step 5: Develop a Costing Plan

#### 3.7.1. Selecting Cost Data Collection Sites

The first step to developing a costing plan is selecting cost data collection sites. Cost data collection sites should be facilities where the EHS modality and level (see step 2) are comparable to how the EHS will be provided in the target facility. Cost data may be collected at the target facility itself. This is most applicable in situations where the target facility is already providing EHS at an adequate level, and the purpose of costing is to budget for sustaining existing EHS, as in Example 1 described in Table 2. However, if the purpose of costing is to budget for the installation of new or major rehabilitation of existing EHS, costing-stakeholders likely must collect cost data from sites other than the target facility. For instance, in Example 3 in Table 2, costing-stakeholders budget for the installation of boreholes and solar pumps at facilities relying on rain and surface water use. To understand the costs of borehole installation, costing-stakeholders cannot visit facilities targeted for EHS delivery but must visit other sites for cost data collection.

When the costs at the target facility are not representative of the costs required to achieve the target outcomes, appropriate alternative cost data collection sites may include comparable facilities of a similar size and type that do provide EHS that meet the target outcomes. Costing-stakeholders may also contact contractors or private suppliers to obtain estimates for EHS delivery at the target facility. When the target facility and cost data collection sites are not the same, costing-stakeholders may need to repeat the contextual assessment in step 4 to ensure that cost data collection sites are comparable to the target facility in terms of facility characteristics, and that EHS at cost data collection sites meet the quantity and quality needs of target facilities.

Similarly, in cases where the number of target facilities is too large to feasibly collect cost data for every facility, costing-stakeholders will need to design a sampling plan of representative facilities. Both the facility context, such as the size and type, and the EHS quantity and quality will influence costs and should be considered when designing a representative sample. Sampling strategies are beyond the scope of this study, and we refer readers to other references [55,56].

Regardless of which facilities are selected, costing-stakeholders will need to secure relevant ethical and administrative permission before data collection. While cost data do not have the same ethical concerns as human subject data, costing-stakeholders will, at a minimum, need to secure permission from facility administrators prior to data collection and should consult with the appropriate local research ethics board to determine if additional approval is needed.

#### 3.7.2. Costing Frameworks

Costing frameworks outline expected expenses based on how an EHS is or will be delivered in the target facility through identifying specific resource inputs. Applied a priori, frameworks help costing-stakeholders to identify expected expenses and ensure that the data collection plan will capture cost information for all resources required for EHS delivery. Applied a posteriori, frameworks can be used to compare expected versus collected costs to assess data gaps (see step 8).

We present an example framework for waste management in Table 4. This framework outlines the activities required for waste management and categorizes the resource inputs required for each activity into the cost categories outlined in Table 3. We developed this example framework through a review of studies captured by the systematic review that describe resources used in waste management activities [39,49,51,57,58,59,60,61,62,63,64,65,66,67,68,69]. We categorized these resources into cost categories, and then cross-referenced selected guidelines for waste management [42,70] to fill gaps. Frameworks could similarly be developed, adapted, or supplemented through primary data collection, for example, through the observation of EHS delivery in selected data collection sites.

The level of detail in a framework required will depend on the costing purpose. Table 4 is designed to facilitate bottom-up costing (see Section 3.7.3). This level of detail may not be necessary for all costing purposes. Where costing is done at a national level using a top-down approach, a framework which simply identifies overall expected costs without an activity-specific breakdown may be appropriate (see, for example, the WHO outline of expenses for waste management [24]). Similarly, costing-stakeholders may not need to account for all activities or cost categories. For example, a small urban clinic that contracts waste treatment and disposal services to a larger facility may categorize these expenses as a contracting fee that bundles all cost categories together, rather than considering each individually.

#### 3.7.3. Costing Approaches

In healthcare, costing approaches are typically either top-down or bottom-up. In top-down costing (TDC), expenditures for services are estimated from an overall budget, where the total budget is apportioned by a unit of analysis, such as hospital days, to assign a cost for a healthcare service [71]. Bottom-up costing (BUC) enumerates the resource inputs for healthcare delivery from records, interviews, or observed use at the HCF level, and estimates the unit costs of service delivery based on the total cost of resource inputs [71,72].

The advantages of TDC are that costs can be estimated from overall budgets and that data collection is less time-intensive than BUC. TDC is well-suited to EHS which are provided through a stand-alone unit within a facility, such as a laundry department or central sterilization unit (see, e.g., [63,68,73,74],). However, a particular challenge of TDC for EHS is determining a unit of assessment. In healthcare settings, TDC might be used to assess the cost of a specific care service (e.g., outpatient services) by dividing the total budget by the number of patients served. However, budgets for EHS costs are often divided across multiple departments and lack comprehensive, consolidated budgets at the facility level [75]. In these cases, TDC may not be feasible. TDC is commonly done retrospectively using records of past funding disbursements but can be done prospectively using planned budgets. However, costing-stakeholders should be careful to consider possible mismatches between planned versus disbursed funding [75].

In contrast, the advantages of BUC are that costs can be collected on individual services within healthcare systems. BUC provides a high level of disaggregated detail on line-item expenses, which may be useful if understanding specific expenditures required for EHS delivery is desirable for the costing purpose [76]. However, BUC can be difficult and labor-intensive to implement [77,78,79]. Bottom-up costing may be done prospectively through observations and surveys, retrospectively through record reviews, or through a combination of both. Record reviews may be less time-intensive but require facilities to maintain accurate records that disaggregate environmental expenses from other costs. Furthermore, BUC may be inappropriate for capturing infrequent but large expenses, such as major infrastructure repairs, which may not be observed during the study period. Longer study periods may ameliorate this risk but are not always feasible given the intensive resource needs of BUC [15].

In LMICs, a barrier to costing healthcare-related services is data availability. Most costing conducted in an LMIC context requires a blend of both BUC and TDC [80]. In the context of costing environmental health services, we recommend costing plans that include a blend of BUC and TDC to ensure that comprehensive data are collected and to enable data verification and validation.

Regardless of the approach selected, costing-stakeholders should pilot tools at the selected data collection sites. Pilot testing may reveal that the chosen approach is inappropriate for the available data sources, and in these instances, costing-stakeholders will need to iterate steps in the planning phase until a feasible approach is identified. Table 2 gives examples of iteration in the planning phase.

### 3.8. Step 6: Identify Data Sources

Possible data sources for costing EHS are diverse and may be collected from internal and/or external sources. Internal sources of data may include interviews with facility staff, and accounting or other financial records. External sources may include bids from contracts, supplier pricing lists, or comparable facilities. In some cases, prices for goods and services paid by public facilities may be fixed at the national level. For example, standard pricing lists for select items in public facilities in Malawi are available (http://cmst.mw/catalogue/).

Data sources and data collection methods are interdependent. Survey tools, structured observation forms, and other questionnaires are appropriate for prospective data collection, while codebooks are appropriate for retrospective record reviews. In many cases, multiple data sources may be necessary to collect the full range of relevant costs, and triangulation across multiple data sources may be useful for evaluating data completeness and accuracy (see step 8). Costing-stakeholders should consider the feasibility of available data sources to execute costing plans and may need to iterate earlier steps in the planning phase if the appropriate data sources are not available.

### 3.9. Step 7: Collect Cost Data

In this step, costing-stakeholders execute their costing plan, collecting data from each relevant data source. If challenges are encountered that make the original plan infeasible, Steps 4–6 of the planning phase should be iterated until a feasible plan is developed. The documentation of all steps in the data collection process for each iteration is important for a later assessment of data quality. The Consolidated Health Economic Evaluation Reporting Standards (CHEERS) statement outlines guidelines for documenting and reporting steps in the costing process, including data sources, approaches for data collection, currency, price data, conversion rates, and any assumptions made [31]. Additionally, we recommend that costing-stakeholders ask individuals responsible for providing or managing cost data to provide a qualitative assessment of data completeness and accuracy. This information is useful for informing data aggregation and evaluation in step 8 and can indicate a need to iterate data collection when the data quality is poor.

### 3.10. Step 8: Aggregate and Evaluate

Data aggregation is necessary when cost data come from different sources and in different formats. A suggested framework for aggregating costs, shown as an example for waste management, is provided in Table 4. When adapting the framework, costing-stakeholders should eliminate any cost categories (columns) determined to be irrelevant during step 3 and list the major activities (rows) required to provide the associated EHS, as identified during step 4. Each cell of the framework can then be filled with any potential expenses identified and associated cost data collected that fall within the scope of relevant costs. Depending on the level of detail required for the costing purpose, data can be aggregated by table or EHS, row or activity, column or cost category, cell, or even further divisions within cells. Aggregating data in this format makes it easier for costing-stakeholders to determine where gaps exist and collect data to fill those gaps.

When costing-stakeholders evaluate cost data for comprehensiveness, they should also assess the quality of the collected data. As a quality check, HCF staff familiar with facility budgets (administrators, accountants, bookkeepers, etc.), as well as staff familiar with departmental budgets (doctors, nurses, cleaners, mechanics, etc.), should confirm that the aggregated cost data reflect their knowledge of internal budgets. Where possible, collecting cost data from multiple sources and triangulating findings to identify discrepancies can also help increase the data quality. However, with multiple sources of data, costing-stakeholders must determine how they will handle conflicting cost data. Conflicting data may indicate true error, such as inconsistent or inaccurate record keeping, or misalignment between budgets and actual spending, such as in cases where budgets allocated for an EHS are insufficient to meet demand and additional funds are sourced elsewhere. Strategies for addressing conflicting data include determining a gold standard data source; seeking guidance from staff identified during quality checks; averaging costs; or selecting the maximum or minimum value, for more or less conservative cost estimates, respectively.

### 3.11. Step 9: Calculate Costs

Calculating the total costs of EHS delivery is, most basically, a function of the amount of total resources required to deliver the EHS, multiplied by the unit cost of each resource. In cases where costing-stakeholders are applying findings to the large-scale funding of multiple facilities, economies of scale may reduce unit costs. Relevant resources may be tangible goods, such as capital hardware expenses, or other services, such as staff wages for personnel or insurance costs for direct support. A common source of error when calculating costs is the omission of “hidden” costs, such as insurance and licensing, which are not readily observable, but may substantially contribute to overall costs [15]. The use of a costing framework, as described above, can help ensure that all relevant costs are included in the calculations.

For accurate calculations, costing-stakeholders must differentiate between the price paid versus the market price. The price paid by a particular HCF may not necessarily reflect all the expenses, such as taxes, procurement, transportation, or insurance, that another facility would need to cover [81]. Similarly, subsidized or donated supplies or uncompensated staff time are often inappropriately considered as “free” because the HCF would not pay for these items, but these goods and services should be included in cost calculations at the market rate to avoid underestimating the true costs of EHS delivery. In some cases, an EHS may have a revenue-generating component, such as the sale of recyclables, resulting in overestimating the costs of EHS delivery [39]. However, costing-stakeholders should be very careful not to overestimate the potential revenues as this will lead to inaccurate underestimates of the cost.

Cost calculations must also appropriately account for resources which are not wholly dedicated to EHS delivery and may serve other purposes or roles in the HCF. In these cases, costing-stakeholders should only include the relevant portion of expenses which are devoted to the EHS. For example, groundskeepers may devote 10% of their time to the maintenance of an HCF’s sanitation facilities, in which case, 10% of their salary should be counted as a relevant cost. This approach is also appropriate for considering building or land use, when rent or other maintenance is a factor, as well as any other indirect costs that are necessary for ensuring that the new facilities or services are provided satisfactorily. However, for cases where a needed resource would not be covered by other sources, the entire costs should be included. For example, if an HCF does not have a groundskeeper, but one is required for delivering a particular EHS, the entire salary should be included as a relevant cost [81,82].

When projecting budgets for future expenses, costing-stakeholders should consider potential uncertainty in costs. One approach to addressing uncertainty is to use a three-point estimating approach which averages “best case, the most likely, and the worst case” scenarios, either as a simple average or a weighted average where the most likely scenario is weighted more heavily [83]. Another approach is to include contingency funds in the estimate of any project, which is often around ten percent of the total project cost [83,84]. For a more detailed introduction to budgeting, see [82] or [81].

Additional considerations may be needed for specific costing purposes. For example, one costing purpose could be to help support securing financial investments or loans. This approach would need to adhere to relevant accounting standards that are different in each country, and costing-stakeholders must ensure that they collect the costs required to satisfy the needs of their costing purpose. Specific calculations are needed in cost-effectiveness or benefit-cost analysis, which are beyond the scope of this study. For these applications of cost data, we recommend that costing-stakeholders include an economist. For a more detailed introduction to benefit-cost analysis and cost-effectiveness analysis, see [40,85], or for relevant examples, see [20,86].

#### Unit Costs

After calculating the total costs, costing-stakeholders must consider unit costs relevant to the purpose and application of data. Units contextualize cost findings and facilitate comparisons across facilities. At a minimum, cost units should include the currency and time frame in which costs were incurred, and any foreign exchange rates used [31]. For retrospective studies, the year in which data were collected will likely not match the year in which costs were incurred, and expenses may need to be adjusted for inflation. Except for capital hardware costs, which are more likely to be a one-time expense, most operation and maintenance costs are measured as a function of time (e.g., costs per year or per equipment lifespan). Costs that are unevenly distributed over the lifecycle of an EHS can be annualized by assessing the total lifecycle costs and dividing them over an anticipated equipment lifespan [18].

Other units, which are useful for comparing costs across facilities, include direct measures or indicators of EHS quantity (see Section 3.6). For example, unit costs per kilogram of waste produced or per patient served are more useful for comparing operation costs of facilities than facility-wide operation costs. Costing-stakeholders should consider how well indicators are likely to correlate with the EHS demand, because the same indicators may not be appropriate across all EHS. For example, floor area is commonly used as an EHS quantity indicator, which correlates more directly with, and is more appropriate for measuring, the demand for surface cleaning [52] than for waste management, which correlates more with patient volume [39,49,50,51]. Costing-stakeholders should select units which are appropriate for their costing purpose and target outcomes.

### 3.12. Step 10. Share and Apply

When sharing cost findings, we recommend that costing-stakeholders adhere to reporting guidelines for economic research [31]. Findings for dissemination comprise more than just cost data. Information on the scope of costs (step 3), facility context (step 4), data collection plan and process (steps 5–7), and any limitations identified when aggregating and evaluating data (step 8) are important for the accurate and appropriate application of data and should be disseminated with cost findings. Sharing findings internally and reviewing with costing-stakeholders that include a diversity of staff from target facilities and data collection sites can help identify limitations and correct potential errors before findings are more widely disseminated and applied.

Measures of cost are important for measuring and achieving successful implementation [87]. The application and sharing of findings will differ, depending on the costing purpose. Cost data may be used at the facility level to inform planning and budgeting. To create a total budget, costing-stakeholders aggregate the cost estimates for each component of the EHS on an annual basis over the lifecycle of the project, adjusting for inflation in each year [81]. The budget can then be presented on this annual basis. Once this budget is allocated and the funds are received by the appropriate purchasing agent, the funds can be allocated. When faced with misalignment between allocated funds and true costs of EHS operation, purchasers should alert project management to transparently communicate potential cost overruns. Routine monthly reviews of budgets can also help identify other potential misalignments between budgeted, allocated, or actual EHS costs [88].

Cost data may also be used to prioritize investment in cost-effective services. If the costing purpose is for a benefit-cost or cost-effectiveness analysis, costing-stakeholders must also identify the benefits or targeted effectiveness outcomes of the analyses. Methods for estimating EHS benefits and effectiveness are beyond the scope of this study, and we recommend that costing-stakeholders consult additional references (e.g., 19, 27, 28) and include stakeholders with relevant expertise from the start of step 1 of the model, to ensure that costs and contextual data collected are fit-for-purpose.

Finally, we recommend that costing-stakeholders make their data publicly available when possible. A lack of rigorous costing evidence impedes progress towards achieving universal access, and systematic reviews indicate a paucity of high-quality evidence [15]. As cost data do not contain confidential patient information, they may be collected and shared on open-access platforms to improve available evidence and inform better decision making [15,89].

### 3.13. Application of this Model Toward Future Research

We intend for this model to be applied for budgeting in a variety of HCF settings in LMIC contexts, from small, rural clinics to large, urban hospitals. However, we anticipate that the specific actions taken in each step will vary by context, and we encourage future research to document the budgeting process, noting any context-specific adaptations.

We encourage costing-stakeholders to apply this model to generate evidence on the costs of EHS in HCFs in LMICs, which can be used to inform budgeting world-wide. Systematic reviews have identified a lack of robust evidence on costs. Estimated costs for achieving various service levels, for example, 10-year costs to install and deliver water at JMP-limited versus basic levels, are largely unknown [15]. Future research can apply this model to generate evidence to inform those estimates and advance progress towards universal access.

In this paper, we identify opportunities to improve indicators and measurement tools for EHS in HCFs. Future research to improve existing or develop new indicators and measurement tools for EHS quality and quantity would improve the reporting of context for more informed budgeting. Similarly, while our model proposes a broad overview of the steps required for budgeting, tools to support actions within each step are lacking. For example, to the best of our knowledge, tools such as structured observation forms or surveys to support bottom-up costing are lacking, and research on how to develop such tools would facilitate future budgeting efforts.

## 4. Conclusions

We developed a ten-step process model to guide budgeting for EHS in HCFs, including steps for the planning, data collection, and synthesis of cost data and other non-cost information needed to contextualize costs. We have presented our model, with the steps numbered and ordered for simplicity. However, in reality, the steps required for budgeting may not be linear and will commonly require iteration. Steps are interdependent, such that to execute cost data collection successfully, several iterations of planning may be required to identify a feasible costing plan and appropriate data sources to match. Rather than aspiring to a linear approach, we encourage costing-stakeholders to embrace the iterative nature of budgeting in order to generate meaningful cost findings that carefully consider the context of EHS delivery and limitations of estimated costs.

Our model is designed for costing-stakeholders to budget for EHS in a variety of HCF settings. However, we caution that the studies reviewed to develop this model costed EHS at the facility- or sub-facility level in LMICs. Most studies we reviewed were conducted in urban hospitals offering inpatient services. We encourage costing-stakeholders to adapt their data collection process as necessary to ensure that methods are feasible in their target facilities and data are fit-for-purpose. We did not review studies conducted in high-income settings or studies evaluating costs at the health systems level, and we did not develop our model to be applied for budgeting in these settings. Facility-level data may inform decisions for funding health systems, but health system funding has many other considerations that are beyond the scope of our model.

We propose the application of this model to address challenges with low-rigor and inconsistent costing of EHS in HCFs [15]. Improving the understanding of costs of EHS delivery can improve the sustainability of existing EHS and accelerate progress towards universal access [90], which are critical for ensuring a safe and hygienic environment for patients, caregivers, and healthcare workers. Cost data can be used for a variety of purposes, from budgeting to planning investment in cost-effective EHS services. In either case, planning and executing data collection to understand costs of EHS in HCFs is a multi-step process that requires more than just cost data. Defining target outcomes, selecting appropriate scope for costs, developing a rigorous costing plan, and identifying data sources are all steps that must be carefully considered before data collection begins. Additionally, collecting data on EHS quantity and quality in parallel with costs is essential for contextualizing cost findings and generating information that can be meaningfully applied to budgeting in other contexts.

## Figures and Tables

**Table 1:** Actions for each costing step in a process model for costing environmental health services (EHS) in healthcare facilities.

	Costing Step	Actions
**Planning**	1. Define costing purpose	Determine the time and resources available for costing Consult stakeholders to agree a costing purpose and application of data Identify outcomes targeted by spending (e.g., reduce healthcare-acquired infections, meet Joint Monitoring Program (JMP) basic sanitation guidelines) Identify a location, target facilities, and a timeframe for target outcomes
	2. Identify relevant EHS	Identify which EHS will be the focus of costing activities Determine relevant modality/ies of EHS provision (e.g., waste management through incineration) Determine relevant EHS provision level(s) (e.g., JMP basic-level waste management)
	3. Define the scope of relevant costs	Define the scope of relevant costs. Consider both technology lifecycles and cost categories. Justify any exclusions of scope as per costing purpose
	4. Collect non-cost contextual data	Describe facility characteristics (e.g., size, patient volume, services provided) Evaluate EHS quantity and quality, and identify any differences between actual and aspirational EHS quantity and quality Assess how EHS are provided. Document EHS inputs and outputs
	5. Develop a costing plan	Develop a costing framework that identifies expected expenses based on EHS inputs and outputs identified in step 4 Select cost data collection sites ^†^ Develop a costing plan that identifies the approach (e.g., top-down, bottom-up, hybrid) and data collection tools Secure relevant ethical and administrative permission Pilot test tools at selected data collection sites. Revise as necessary
	6. Identify data sources	Identify key informants and records systems for data collection. Informants may be internal to the facility or external (e.g., contractors or construction firms) Assess the feasibility of the costing plan from Step 4 based on available data sources. Revise as necessary.
**Data collection**	7. Collect cost data	Execute the costing plan from Step 5. Collect data from all relevant data sources identified in step 6 Document each data source and iteration (see step 8) Ask relevant stakeholders to evaluate data quality and completeness for each source
	8. Aggregate and evaluate	Aggregate data from all sources and categorize into the costing framework Compare expected versus documented costs and identify data gaps Iterate steps 6–8 to fill these gaps
**Synthesis**	9. Calculate costs	Calculate total costs. Adjust for taxes, subsidies/tariffs, financing costs, deprecation, or other factors as needed Calculate relevant unit costs Conduct sensitivity analysis (e.g., identify potential variance in costs, inflation)
	10. Share and apply	Adhere to reporting guidelines for economic studies^‡^ Share and review with internal stakeholders as a final validity check Disseminate more widely to external stakeholders. Make data publicly available when possible. Plan for updating information systems, recurrent data collection, learning

† Cost data collection sites may be target facilities selected in step 1 or other facilities. Where aspirational and actual EHS levels do not match in target facilities, costing-stakeholders will need to identify alternative sites for cost data collection where EHS are provided at aspirational levels.

**Table 2:** Hypothetical examples of the planning stage for different budgeting.

	Example 1: Operating and Maintaining Existing Services	Example 2: Rehabilitating Inadequate Services	Example 3: Installing New Services
Setting	District hospital establishing an annual operations and maintenance budget for waste management	Government of Cambodia budgeting for facility upgrades in fiscal year 2021/2022	Non-governmental organization constructing improvements in a small network of private facilities
Define costing purpose	**Target facility and outcome:** Safely operate and maintain waste disposal equipment in a district hospital in urban Maputo for one year	**Target facility and outcome:** Rehabilitate sanitation facilities to meet WHO guidelines for menstrual hygiene management and disabilities access for all health posts in three rural provinces of Cambodia	**Target facility and outcome:** Provide access to basic water for drinking and care provision for 10 years at five clinics in rural Zambia currently relying on surface water and intermittent rainwater harvesting
Identify relevant EHS	**Relevant EHS:** Waste management **Modality:** Incinerator, waste pits **Service level:** Compliance with World Health Organization (WHO) guidelines for safe management of infectious and sharps waste ^1^	**Relevant EHS:** On-site sanitation **Modality:** Pit latrines **Service level:** Improved facilities meeting WHO guidelines for menstrual hygiene management and disability accessibility ^2^	**Relevant EHS:** On-site water source **Modality:** Boreholes with solar pump, to be connected to existing rooftop rainwater harvesting containers and gravity-fed distribution system **Service level:** JMP basic water
Define the scope of relevant costs	**Lifecycle:** Operations and maintenance for 1 year **Cost categories:** Capital maintenance, consumables, personnel, and direct support **Excluded from scope:** Capital hardware, capital software, and financing costs excluded because infrastructure is already established	**Lifecycle:** Installation of new or upgraded latrines; disposal or demolition of existing latrines **Cost categories:** Capital hardware, capital software, financing **Excluded from scope:** Capital maintenance, consumables, recurrent training, personnel, and direct support, as operations and maintenance covered under separate financing stream	**Lifecycle:** Installation, operation, and maintenance for 10 years **Cost categories:** Capital hardware, capital maintenance **Excluded from scope:** Capital software and recurrent training as boreholes are common locally and staff already know how to use them; consumables as guidelines for basic service do not require testing
Collect non-costs contextual data	Assess Joint Monitoring Program (JMP) indicators for waste management and compliance with WHO guidelines in step 1. Qualitatively document waste disposal equipment, common maintenance requests, consumables, and staff training for operation of equipment	Design a random sample of health post, stratified by province. Assess JMP indicators for sanitation and WHO indicators for disability and menstrual hygiene accessibility. Assess rehabilitation needs, and identify major repair types	Document existing water infrastructure in target clinics. Assess demand for water based on patient volume and type of service provided
Develop a costing plan	**Costing framework:** Categorize inputs identified in step 4 into facility-specific framework **Cost data collection sites:** Target facility **Approach:** Hybrid top-down and bottom-up costing **Data collection tools:** Identify key informants, utilize existing budgets and records from maintenance department, develop surveys to assess consumable and personnel costs	**Costing framework:** Develop frameworks for expected expenses for each major repair type **Cost data collection sites:** Purposively sample 2 and 3 facilities to match major repair type for costing **Approach:** Bottom-up **Data collection tools:** Develop surveys to assess resource inputs and unit costs to rehabilitate latrines	**Costing framework:** Identify expected expenses based on local borehole installation practices **Cost data collection sites:** Select facilities with basic services comparable to target clinics **Approach:** Top-down costing **Data collection tools:** Past budgets or contract bids for borehole installation; codebooks to identify and apportion relevant expenses
Identify data sources	Verify existence of budgets for maintenance for top-down costing Pilot test surveys for bottom-up costing	Identify maintenance and construction workers knowledgeable about rehabilitation costs. Supplement with external contractors as needed	Verify that records exist to facilitate top-down costing
Sample iteration in planning phase	Data sources for top-down costing are unavailable (no budgets records kept). Account for equipment and parts needed for maintenance through bottom-up costing. Verify through contacting external maintenance contractors	Facilities selected during purposive sampling do not match repair types. Resample to identify appropriate facilities	Data sources for top-down costing are unavailable (no records kept) at data collection sites. Contact local contractors for estimates of borehole installation

^1^ WHO guidelines described in the safe management of wastes from health-care activities [42]; ^2^ Improved facilities consisting of superstructures with a roof and door, locks, washing capabilities, menstrual product disposal, access without stairs/steps, and handrails.

**Table 3:** Definitions of cost categories for environmental health services (EHS) in healthcare facilities.

Cost Category	Definition
Capital hardware	Infrastructure or equipment purchases required to establish services or implement changes to EHS delivery method, which are not consumed during normal EHS operation
Capital maintenance	Expenses required to repair, rehabilitate, or otherwise maintain functionality of capital hardware, including labor costs required for these purposes
Capital software	Planning, procurement, and initial training costs associated with establishing new services or implementing changes to EHS delivery method
Recurrent training	Training required to ensure proper ongoing EHS provision, regardless of changes to EHS delivery
Consumables	Products and supplies that are consumed during normal operation
Personnel	Labor costs associated with normal operation of a service, including staff benefits
Direct support	Expenses required to supervise and monitor EHS provision to ensure safety and sustainability that support, but do not have direct EHS outputs, such as auditing or developing management plans
Financing	Loan interest and other fees associated with EHS financing
Contracted services	Fees paid to external providers to perform all or part of normal EHS operation, including multiple other cost categories, where expenses cannot be accurately disaggregated into categories above; where fees fall solely within another cost category described above, expenses should be included therein

**Table 4:** Waste management framework in healthcare facility life cycle costing.

Activity	Expense Category ^†^								
	Capital Hardware	Capital Maintenance	Capital Software	Recurrent Training	Consumables	Personnel	Direct Support	Financing	Contracted Services
Collection, segregation, packaging, and storage	Point-of-use waste receptacles; interim bulk storage containers; syringe/needle cutters; storage room/area; storage area refrigeration units; waste weighing scale	Cleaning and disinfection of collection and waste storage containers and areas; storage area repairs and maintenance	Equipment operation training; design consultations: environmental/waste management, engineering, and architectural design	Sharps and hazardous waste safe handling and disposal training	Disposable waste containers (sharps bins, biohazard bags, etc.); personal protective equipment; waste labeling materials; cleaning and disinfection equipment; spare parts and tools for equipment maintenance and repairs	Point-of-care providers (nurses, technicians, etc.); support staff; cleaners	Compliance monitoring and audits; immunizations for waste handlers	Taxes; interest; utility costs (water, electricity, fuel, etc.); labor and installation fees for equipment	Extra fees or expenses paid to external contractor for any part of the collection, segregation, packaging, and storage process
Transportation: pre- and post-treatment	Trolleys, carts, or other equipment for on-site transport; transportation containers; transportation vehicles for off-site transport	Cleaning and disinfection of waste storage containers and transportation equipment; vehicle repairs and maintenance	Equipment operation training; design consultations: environmental/waste management, engineering, and architectural design	Sharps and hazardous waste safe handling and disposal training; safe transport training	Disposable waste containers; personal protective equipment; waste labeling materials; cleaning and disinfection equipment; spare parts and tools for equipment maintenance and repairs; vehicle fuel	Loading/unloading staff; drivers; support staff	Vehicle licensing and insurance; compliance monitoring and audits; immunizations for waste handlers	Taxes; interest	Extra fees or expenses paid to external contractor for any part of the transportation of waste
Treatment: incineration, microwaving, autoclave/hydroclave, chemical disinfection, steam sterilization, or dry thermal^ ‡^	Solid waste treatment equipment; liquid waste treatment equipment; waste shredders; pollution control on incinerators	Cleaning and disinfection of treatment equipment and treatment area; treatment equipment repairs and maintenance	Equipment operation training; design consultations: environmental/waste management, engineering, and architectural design	Sharps and hazardous waste safe handling and disposal training	Disposable components of treatment process; treatment chemicals; personal protective equipment; cleaning and disinfection equipment; spare parts and tools for equipment maintenance and repairs	Treatment plant staff	Treatment plant licensing; incineration air quality emissions testing; compliance monitoring and audits; immunizations for waste handlers	Taxes; interest; utility costs; labor and installation fees for equipment	Extra fees or expenses paid to external contractor for any part of the waste treatment process
Final disposal: solid waste landfilling, liquid waste discharge	Landfill/disposal site; sewerage system	Landfill/disposal site repairs and maintenance; sewerage repairs and maintenance	Design consultations: environmental/waste management, engineering, and architectural design	Sharps and hazardous waste safe handling and disposal training	Personal protective equipment	Loading/unloading staff; treatment plant staff	Landfill licensing; compliance monitoring and audits; immunizations for waste handlers	Taxes; interest; landfill fees for solid waste; sewerage fees for liquid waste	Extra fees or expenses paid to external contractor for any part of the final waste disposal process

† Some activities and/or expenses may be considered under multiple categories. For example, the cleaning and disinfection of waste areas may be considered a maintenance expense, but also requires consumable products. Costing-stakeholders may classify expenses in either cost category as appropriate for their context and costing purpose but should be careful not to double-count expenses in multiple categories. ‡ Treatment row outlines expenses for multiple possible treatment options (e.g., chemical or incineration). Costing-stakeholders should consider only expenses that are relevant for the treatment method used at their target facility. Not all expenses will apply (e.g., pollution control for incinerators only applies to facilities using incineration for waste treatment).

## Supplementary Materials

The following are available online at https://www.mdpi.com/1660-4601/17/6/2075/s1: Table S1: bibliographic information of studies informing model development.
